# Improving the Stability of Protein–Protein Interaction Assay FlimPIA Using a Thermostabilized Firefly Luciferase

**DOI:** 10.3389/fbioe.2021.778120

**Published:** 2021-11-11

**Authors:** Yuki Ohmuro-Matsuyama, Keiko Gomi, Takuya Shimoda, Hideki Yamaji, Hiroshi Ueda

**Affiliations:** ^1^ Laboratory of Chemistry and Life Science, Institute of Innovative Research, Tokyo Institute of Technology, Yokohama, Japan; ^2^ Department of Chemical Science and Engineering, Graduate School of Engineering, Kobe University, Kobe, Japan; ^3^ Technology Research Laboratory, Shimadzu Corporation, Kyoto, Japan; ^4^ Kikkoman Co., Ltd., Noda, Japan

**Keywords:** protein-protein interaction (PPI), firefly luciferase (Fluc), bioluminescence, adenylation, oxidative reaction, thermostability, assay stability

## Abstract

The protein–protein interaction assay is a key technology in various fields, being applicable in drug screening as well as in diagnosis and inspection, wherein the stability of assays is important. In a previous study, we developed a unique protein–protein interaction assay “FlimPIA” based on the functional complementation of mutant firefly luciferases (Fluc). The catalytic step of Fluc was divided into two half steps: D-luciferin was adenylated in the first step, while adenylated luciferin was oxidized in the second step. We constructed two mutants of Fluc from *Photinus pyralis* (Ppy); one mutant named Donor is defective in the second half reaction, while the other mutant named Acceptor exhibited low activity in the first half reaction. To date, Ppy has been used in the system; however, its thermostability is low. In this study, to improve the stability of the system, we applied Fluc from thermostabilized *Luciola lateralis* to FlimPIA. We screened suitable mutants as probes for FlimPIA and obtained Acceptor and Donor candidates. We detected the interaction of FKBP12-FRB with FlimPIA using these candidates. Furthermore, after the incubation of the probes at 37°C for 1 h, the luminescence signal of the new system was 2.4-fold higher than that of the previous system, showing significant improvement in the stability of the assay.

## 1 Introduction

**FIGURE 1:**
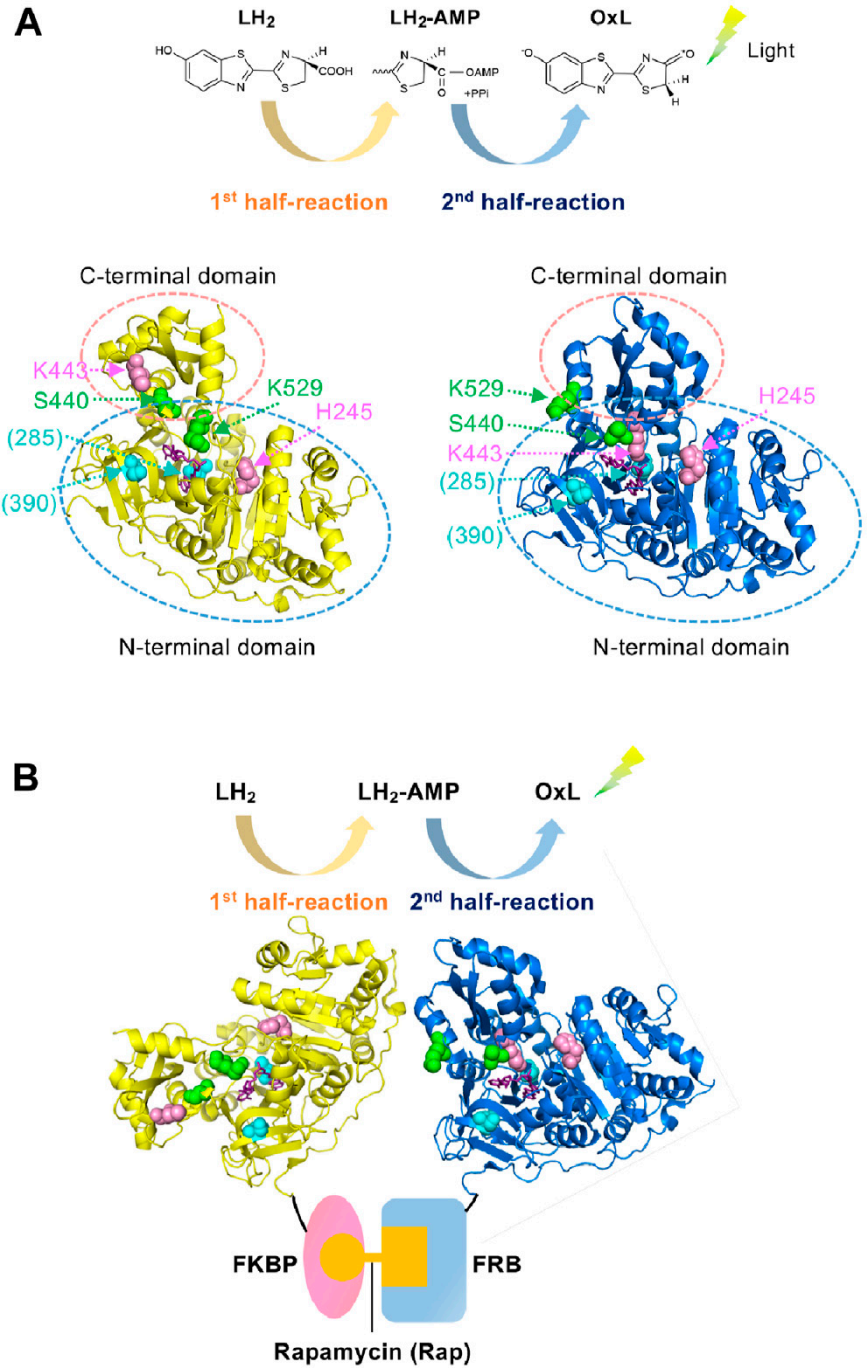
Working principle of FlimPIA **(A)** Chemical reaction catalyzed by Fluc. Each of the two half-reactions is catalyzed by the different conformation. Ppy enzyme at the adenylation conformation (pdb 4G36) is shown in yellow, while that for the oxidative light-emitting reactions (pdb 4G37) is shown in blue. Magenta: the mutated residues in the Donor; green: those in the Acceptor; cyan: sites mutated for improved stability in LlL (in parentheses); purple; substrate analogue DLSA. The residue numbers are shown as those in Ppy throughout. **(B)** The scheme of FlimPIA in this paper. When the Donor is far from the Acceptor, LH_2_-AMP produced by the Donor does not reach the Acceptor. When the PPI between FKBP and FRB is induced by rapamycin, the Donor and Acceptor are in close proximity and the Acceptor catalyzes LH_2_-AMP produced by the Donor more efficiently.

The protein–protein interaction (PPI) assay is a key technology, not only in basic biology but also for practical purposes in drug screening (Gul and Hadian, 2014; Xu et al., 2016; Ashkenazi et al., 2017), diagnosis (Yuan et al., 2017; Atan et al., 2018), and food inspection (Rasooly, 2001; Katam et al., 2016). We previously developed a PPI assay based on the two divided catalytic reactions of firefly luciferase (Fluc); the first catalytic half-reaction involves the adenylation of D-luciferin (LH_2_), which produces the reaction intermediate, luciferyl adenylate (LH_2_-AMP), while the second half-reaction involves the oxidation of LH_2_-AMP, which produces oxyluciferin (OxL). Subsequently, the excited OxL emits light (Figure 1A) (Branchini et al., 2011; Sundlov et al., 2012). Two Fluc mutants named Donor and Acceptor were made of Fluc from *Photinus pyralis* (Ppy). The Donor maintains the activity in the first half-reaction but is defective in the second half-reaction, while the Acceptor has a low activity in the first half-reaction but mostly retains the activity in the second-half reaction. Therefore, the Donor can produce the reaction intermediate, LH_2_-AMP, while producing almost no OxL. However, the Acceptor cannot produce LH_2_-AMP but can retain the catalytic activity for the oxidation of the intermediate. When the Donor and Acceptor are each fused to an interacting protein, the interaction of the proteins induces the approximation of the two mutant enzymes. Based on this principle, previously, we succeeded to detect 1) the interaction between FK506-binding protein 12 (FKBP12) and FKBP-rapamycin-associated protein (FRB), which interact depending on an immunosuppressant rapamycin, and 2) that between p53 and its inhibitor Mdm2, which controls apoptosis, senescence, DNA repair and cell growth. Each PPI results in a higher rate of LH_2_-AMP catalysis into OxL by the Acceptor, leading to the higher light emission (Figure 1B). This PPI assay was named Firefly luminescent intermediate Protein-protein Interaction Assay or FlimPIA (Ohmuro-Matsuyama et al., 2013b; Ohmuro-Matsuyama et al., 2014; Kurihara et al., 2016; Ohmuro-Matsuyama and Ueda, 2016, 2017; Ohmuro-Matsuyama et al., 2018a; Ohmuro-Matsuyama et al., 2018b).

FlimPIA has several advantages. The signal/background ratio and sensitivity are higher, and the detectable size of the interacting protein is larger than that of fluorescent protein-based Förster/fluorescence resonance energy transfer assay and Fluc-based protein-fragment complementation assay. Furthermore, the thermostability of the probe is 4-fold higher than that of Fluc PCA probes because of the unstable split forms of PCA probes and the structural integrity of FlimPIA probes employing the full-length Fluc (Ohmuro-Matsuyama et al., 2013a).

However, the thermostability of FlimPIA remains insufficient owing to the low stability of Ppy. Although Fluc is applied to a hygiene monitoring system (Corbitt et al., 2000; Bakke and Suzuki, 2018), incubation of the enzyme for 1 h at 37°C resulted in an 83% decrease in signal intensity. To overcome this problem, many researchers have attempted to enhance the stability of Fluc (White et al., 1996; Koksharov and Ugarova, 2011a; Koksharov and Ugarova, 2011b; Rasouli et al., 2011; Lohrasbi-Nejad et al., 2016; Solgi et al., 2016; Branchini and Southworth, 2017; Jazayeri et al., 2017; Rahban et al., 2017; Pozzo et al., 2018; Branchini et al., 2019). In this study, we applied *Luciola lateralis* (Heike-botaru) Fluc (LlL) with mutations for thermostability and organic solvent tolerance (Kodama and Nasu, 2011) to FlimPIA for improving the stability at physiological reaction condition. LlL was obtained by random mutagenesis and screening of a high activity mutant L344A, encoding two mutations V287A and V392I.

## 2 Materials and Methods

### 2.1 Materials

Firefly luciferin (LH_2_) was obtained from Biosynth AG (Staad, Switzerland). Rapamycin was purchased from LKT Laboratories (St. Paul, MN, United States), and 3-(N-morpholino) propanesulfonic acid (MOPS) was purchased from Dojindo (Kumamoto, Japan). Oligonucleotides were synthesized by Eurofins (Tokyo, Japan). A QuikChange Site-Directed Mutagenesis Kit was obtained from Agilent (Santa Clara, CA, United States). An In-Fusion HD Cloning Kit was purchased from Takara-Bio (Shiga, Japan). Restriction enzymes and *E. coli* SHuffle T7 express lysY were obtained from New England Biolabs (Ipswich, MA, United States). *E. coli* JM109 competent cells were from SciTrove (Tokyo, Japan). Overnight Express Autoinduction System and BugBuster Master Mix were obtained from MERCK (Kenilworth, NJ, United States). Other reagents of the highest grade available were purchased from Kanto Chemicals (Tokyo, Japan), Fujifilm-Wako Pure Chemical Industries (Osaka, Japan), or Nacalai-Tesque (Kyoto, Japan), unless otherwise indicated.

### 2.2 Construction of Plasmids and Their Libraries

The primers used in this study are listed in Supplementary Table S1. To yield pET26-LlL (H245D), QuikChange Site-Directed Mutagenesis Kit was used according to the manufacturer’s protocol using pET26-LlL (Kodama and Nasu, 2011) as a template and the primer set of LlLH245S_BspHI+ and LlLH245D_BspHI+-r. To yield pET26-LlL(K443A), the same procedure was used with the primers of LlLK443A_AseI+ and LlLK443A_AseI+-r. To yield pET-LlL (K529A; the lysine was at 529 in Ppy and 530 in LlL), the primers of LlLK530A_MfeI+ and LlLK530A_MfiI+-r were used, whereas primers of LlL K530Q_AgeI+ and LlLK530Q_AgeI+-r were used to yield pET26-LlL(K529Q).

To yield FK506-binding protein 12 (FKBP12)-fused LlL(K443A), LlL(K443A) was amplified by polymerase chain reaction (PCR) using pET26-LlL(K443A) as a template and the primers LlLNotG4S-for and LlLXho-rev. The amplified fragment was digested with *Not*I and *Xho*I and inserted between the *Not*I and *Xho*I sites of pET32-FKBP-Donor, which was constructed previously (Ohmuro-Matsuyama et al., 2013b). To yield FKBP12-rapamycin complex-associated protein (FRB)-fused LlL (K529Q), LlL (K529Q) was amplified by PCR using pET26-LlL(K529Q) as a template and the primers LlLNotG4S-for and LlLXho-rev. The amplified fragment was digested with *Not*I and *Xho*I and inserted between the same sites of pET32-FRB-Donor.

To acquire the FRB-LlL(S440X/K529Q) library, QuikChange site-directed mutagenesis was performed using pET32-FRB-LlL(K529Q) as a template and LlL447_KpnI and LlLS440X-KpnI-r as primers. Because bulky amino acids such as leucine and phenylalanine at the position of 440 were suitable as the Ppy Acceptor, the primer LlLS440X-KpnI-r was designed to selectively encode these residues at this position. To yield the FRB-LlL(S440M/K529X) library, the full-length pET32-FRB-LlL (S440M/K529Q) was amplified and site-directly mutated via PCR using the primer set of LlL530X-for and LlLS530X-rev. As the primers encoded 15 nt each of homologous sequences at the ends, both ends of the amplified fragment were recombined by In-Fusion cloning. To yield the FKBP-LlL(H245X/K443A) library, the full-length pET32-FKBP-LlL(K443A) was amplified and site-directly mutated via PCR with the primer set of LlLH245X-for and LlLH245X-rev. Both ends of the amplified fragment were recombined using In-Fusion cloning. To obtain the FKBP-LlLH245E/K443A library, pET32-FKBP-LlL(H245E/K443A) was amplified and site-directly mutated via PCR using the primer set of LlLK443X-for and LlLK443X-rev. Both ends of the amplified fragment were recombined using In-Fusion cloning, according to the manufacturer’s protocol. To confirm that each resultant library contained sufficient variation of the mutants, the DNA sequences of several clones were analyzed.

To obtain FRB-LlL(S440F/K529Q) and FRB-LlL(S440W/K529Q), pET32-FRB-LlL (S440M/K529M) was amplified and site-directly mutated by PCR using the primers LlLS440FW-KpnI and LlL447-KpnI-r, and both ends of the amplified fragment were fused by In-Fusion cloning. pET32-FKBP-LlL(H245E/K443A/I530R) and pET32-FKBP-LlL(H245E/K443A/I530K) were obtained similarly using pET32-FKBP-LlL(H245E/K443A) as a template and LlLI530X-for and LlLI530X-rev as primers.

### 2.3 Expression and Characterization of the Probe Proteins

The probes for FlimPIA were expressed in *E. coli* SHuffle T7 Express lysY and purified using the TALON metal affinity resin, as described previously (Ohmuro-Matsuyama et al., 2013b). The purification profile of each protein was confirmed by SDS-PAGE (Supplementary Figure S1). The kinetic constants *K*_m_ and *V*_max_ for LH_2_-AMP were determined using chemically synthesized LH_2_-AMP, as described previously (Kurihara et al., 2016). To estimate the adenylation activity, the amount of enzymatically formed LH_2_-AMP was measured using the N-domain of Ppy Fluc, as described previously (Ayabe et al., 2005).

### 2.4 Detection of PPI by FlimPIA

An equimolar mixture of the Donor and Acceptor candidates with or without rapamycin in the reaction buffer (50 µl of 100 mM MOPS and 10 mM MgSO_4_; pH 7.3), wherein the concentration of MgSO_4_ and pH were previously optimized for FlimPIA (Ohmuro-Matsuyama et al., 2013b), was added to a well of a 96-well half-area white plate (3693, Corning, Tokyo, Japan). The measurement was performed using a microplate-based luminometer AB2350 at 0.1-s intervals (ATTO, Tokyo, Japan) immediately after injecting 50 µl of the reaction buffer containing the substrates (LH_2_ and ATP). Rapamycin was dissolved in dimethyl sulfoxide (DMSO), and the same amount of DMSO (0.1%) was added as a vehicle to the negative sample. In the optimized buffer conditions, 1 mM of coenzyme A was added to reduce the amount of spontaneously produced dehydroluciferyl-AMP (L-AMP), which acts as a competitor of LH_2_-AMP and inhibits light emission. The addition of coenzyme A results in the conversion of L-AMP to dehydroluciferyl-coenzyme A, which does not act as a competitor (Fontes et al., 1997; Fraga et al., 2005; Kurihara et al., 2016).

### 2.5 Screening

*E. coli* SHuffle T7 Express lys Y was transformed with the mutant plasmids and cultured on LBA agar plates (10 g/L tryptone, 5 g/L yeast extract, 5 g/L sodium chloride, 100 μg/ml ampicillin, and 15 g/L agar). The colonies were selected and cultured using the Overnight Express Autoinduction System in a 96-well culture plate at 30°C overnight. The pellet of each clone was lysed in 50 µl of BugBuster Master Mix, according to the manufacturer’s protocol. The lysates were centrifuged, and the supernatants were collected. To estimate the adenylation activity, each supernatant (10 µl) was added to 90 μl of reaction buffer containing 150 µM of LH_2_ and 2 mM ATP. To estimate the oxidative luminescent activity, 90 µl of the reaction buffer containing 200 nM LH_2_-AMP was added to each supernatant (10 µl).

## 3 Results and Discussion

### 3.1 Testing Ppy Mutations in LlL-Based FlimPIA

Previously, two mutant Ppy enzymes were used to perform FlimPIA. Fluc consists of N- and C-terminal domains, and the C-terminal domain rotates ∼140° according to the reaction proceeds from the first to the second half-reaction (Branchini et al., 2011; Sundlov et al., 2012). Based on this knowledge, the H245D/K443A/I530R mutant of Ppy has been used as the Donor that produces luciferyl adenylate (Ohmuro-Matsuyama et al., 2013b). Among the three mutations, two mutations, H245D near the active site (Ayabe et al., 2005) and K443A in the C-terminal domain (Branchini et al., 2005), suppress the oxidative reaction steps, thereby playing a central role as the Donor. In contrast, being K529 as an essential residue for the adenylation reaction (Branchini et al., 2000), Ppy enzymes with the mutations K529A, K529Q, K529Q/S440F, or K529Q/S440W were used as the Acceptors. While the mutations at K529 are most important for the Acceptor that suppress the adenylation reaction, those for S440 located in the hinge region between the N- and C-terminal domains augment the steric hindrance to suppress the adenylation conformation (Ohmuro-Matsuyama et al., 2018b) (Figure 1). Based on these previous observations, we first obtained the LlL mutants of H245D, K443A, K529A, and K529Q and then analyzed their kinetic constants for LH_2_-AMP (Supplementary Table S2). As a result, suppression of the oxidative reaction steps by H245D and K443A mutations was observed. In contrast, the reaction rate was relatively maintained by the K529A and K529Q mutant LlLs. Next, the relative amounts of LH_2_-AMP produced by the same mutant LlLs were compared to analyze the adenylation activity. The amount of adenylate produced by K443A was higher than that produced by H245D, K529A, and K529Q (Supplementary Figure S2). These results indicate that K443A might act as a Donor because of its high adenylation activity and low oxidative activity, suggesting the importance of K443 in the oxidative steps in LlL. However, the adenylation activity of H245D was too low to act as a Donor. Next, FlimPIA using heterogeneous pairs of LlL-based and Ppy-based mutants was performed using 1) the pair of FKBP-fused LlL (K443A) as a Donor and FRB-fused Ppy Acceptor and 2) the pair of FKBP-fused Ppy Donor and FRB-fused LlL(K529Q) as a possible Acceptor (Supplementary Figure S3). Since the interaction between FKBP and FRB is rapamycin (Rap) dependent (Brown et al., 1994; Chiu et al., 1994; Chen et al., 1995), the luminescence intensity was expected to increase with the addition of Rap. As a result, when rapamycin was added to the first pair of LlL(K443A) and Ppy Acceptor, a higher luminescent intensity was observed in the presence of Rap. However, the second pair of LlL K529Q and Ppy Donor did not show a Rap-dependent signal.

### 3.2 Strategy for Screening LlL Mutants

Because the mutations of H245D and K529Q were not functional for LlL-based FlimPIA, we decided to perform screening for more suitable mutants by the following procedure. 1) The saturation mutagenesis libraries, K529X, S440X, H245X, and K443X were screened. *E. coli* was transformed using the library, and several clones were expressed in the cultured *E. coli* using an autoinduction system. 2) To analyze the first half reaction, ATP and LH_2_ were added to the lysates of *E. coli*. 3) To assess the second half reaction, LH_2_-AMP was added to the lysates. 4) The ratios of the luminescence intensity of 2) and that of 3) were calculated. When the ratio was high, the clone was suitable as a Donor, whereas when the ratio was low, the clone was suitable as an Acceptor.

### 3.3 Screening for LlL Acceptor

**FIGURE 2:**
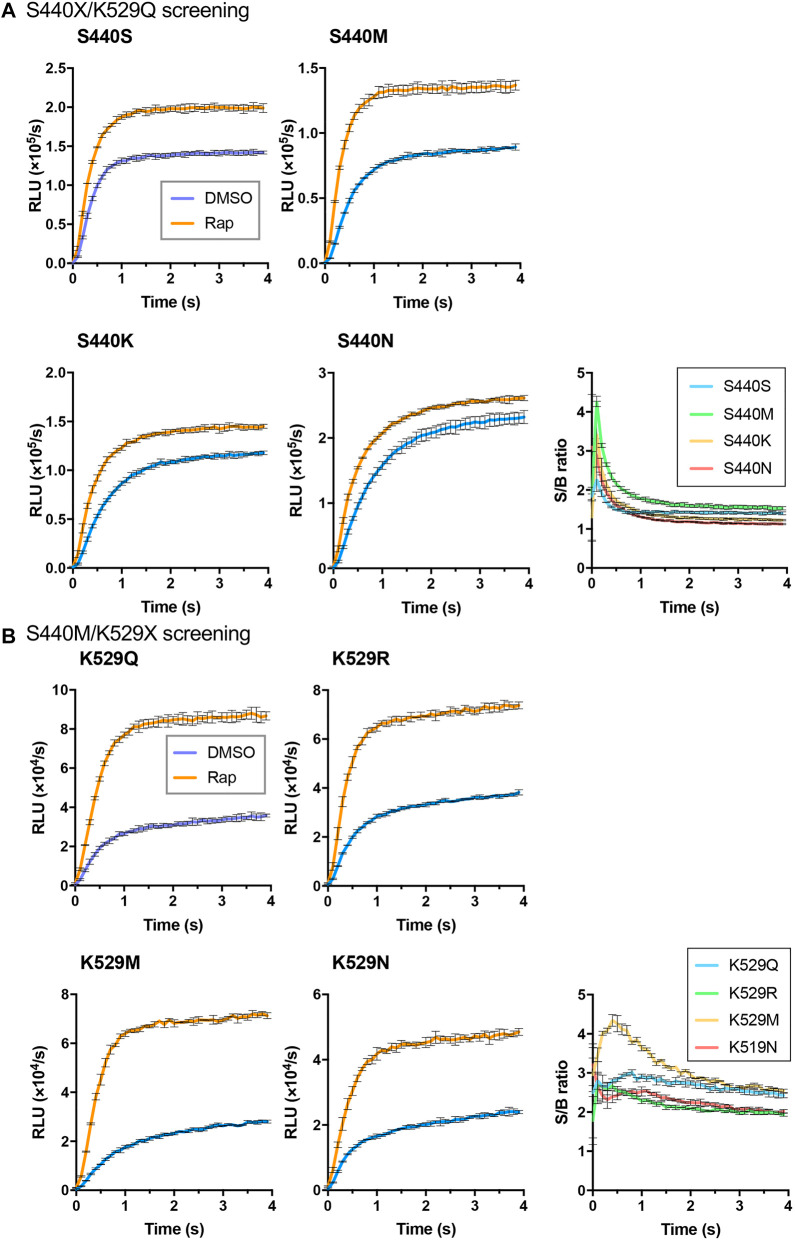
Screening of LlL Acceptors. **(A)** S440X/K529Q screening. **(B)** S440M/K529X screening. Error bar: ±1 × SD (*n* = 3). Ppy Donor (50 nM) and each candidate of LlL Acceptor (50 nM) were reacted with ATP (10 mM) and LH_2_ (37.5 μM) with/without rapamycin (Rap) (50 nM).

As the interacting proteins, FKBP and FRB were used, and the rapamycin dependent interaction was detected (Figure 1B). To obtain an LlL*-*based Acceptor, an FRB/S440X/K529Q library was created, and 96 colonies were selected for screening. Among them, five clones, namely, FRB/S440N/K529Q (two clones), FRB/S440K/K529Q (two clones), and FRB/S440M/K529Q, were selected. The proteins of FRB/S440S/K529Q, FRB/S440N/K529Q, FRB/S440K/K529Q, and FRB/S440M/K529Q were expressed in *E. coli* and purified. Using each protein as the Acceptor and FKBP/Ppy Donor, FlimPIA was performed (Figure 2A). The luminescence intensities of all Acceptor candidates were increased by the addition of Rap. The maximum signal/background (S/B) ratios were in the order of S440M>S440K>S440N>S440S.

In our previous study (Ohmuro-Matsuyama and Ueda, 2017), Ppy (S440F/K529Q) and Ppy (S440W/K529Q) were found to be suitable as the Acceptors. To validate the results of the screening in this study, FRB/LlL(S440F/K529Q) and FRB/LlL(S440W/K529Q) were also prepared and tested. FlimPIA was performed using each FRB/LlL Acceptor candidate and FKBP/Ppy Donor. When Rap was added, the maximum S/B ratios of S440F/K529Q and S440W/K529Q were lower than those of S440S/K529Q (Supplementary Figure S4). Previously, we showed that the optimization of ATP concentration was important for the suppression of the background luminescence intensity and the enhancement of the S/B ratio. Although different concentrations of ATP (20, 5, and 1.25 mM) were examined, the maximum S/B ratios remained low. Therefore, we concluded that the screening method was suitable for the selection of mutants for FlimPIA.

To obtain a better mutant Acceptor, the FRB/LlL S440M/K529X library was constructed. In addition, 6 out of 192 clones were selected. The truncated Flucs were observed in two clones, and the remaining clones were FRB/S440M/K529R (two clones), FRB/S440M/K529M, and FRB/S440M/K529N. FlimPIA was performed using each FRB/LlL Acceptor candidate and FKBP/Ppy Donor. As a result, all candidates acted as the Acceptors, and the maximum S/B ratio of K529M was remarkably higher than that of the other candidates (Figure 2B).

### 3.4 Screening for LlL Donor

To obtain an LlL-based Donor, screening was performed using the FKBP/LlL H245X/K443A library. As a result, 9 out of 288 clones were selected. These included FKBP/H245N/K443A (three clones), FKBP/H245E/K443A (three clones), FKBP/H245A/K443A, FKBP/H245S/K443A, and FKBP/H245P/K443A. FlimPIA was performed using each candidate and FRB/LlL(S440M/K529M). Among these candidates, only H245E/K443A showed increased luminescence intensity upon Rap addition (Supplementary Figure S5). However, the S/B ratio and luminescence intensity were significantly lower than those obtained with the FKBP/Ppy Donor and FRB/LlL(S440M/K529M).

To improve the LlL Donor, another screening was performed using the FKBP/LlL (H245E/K443X) library. Among 288 clones, H245E/K443R (three clones), H245E/K443G (two clones), H245E/K443V, H245E/K443P, and H245E/K443A were selected. FlimPIA was performed using each candidate and the FRB/Ppy Acceptor (K529Q/S440F) (Supplementary Figure S6A). However, the results using these candidates were almost the same for several ATP concentrations. In addition, FRB/LlL (H245E/K443A/I530R) and FRB/LlL (H245E/K443A/I530K) were expressed in *E. coli* and purified. Because I530R is effective for the stabilization of ATP in Ppy (Fujii et al., 2007), we hypothesized that the positively charged amino acids at this position (I530R, I530K) might enhance the adenylation activity. However, in FlimPIA, these mutations decreased the luminescence intensities and could not increase the S/B ratios under several ATP concentrations (Supplementary Figure S6B).

### 3.5 FlimPIA Using Selected Enzymes

Because of the low intensity and S/B ratio of the FKBP/LlL Donor (H245E/K443A) and FRB/LlL Acceptor (S440M/K529M), the stability of the heterologous pair of the Ppy Donor and LlL Acceptor was compared with that of the LlL Donor and LlL Acceptor pair (Supplementary Figure S7). FlimPIA was performed after two overnight incubations at 25°C. Unexpectedly, the S/B ratio and luminescence intensity of the heterologous pair were as high as those of the homologous pair. Ppy Donor might have produced an excess amount of LH_2_-AMP for FlimPIA; therefore, the lower production of LH_2_-AMP after the incubation might not remarkably change the result of FlimPIA.

**FIGURE 3:**
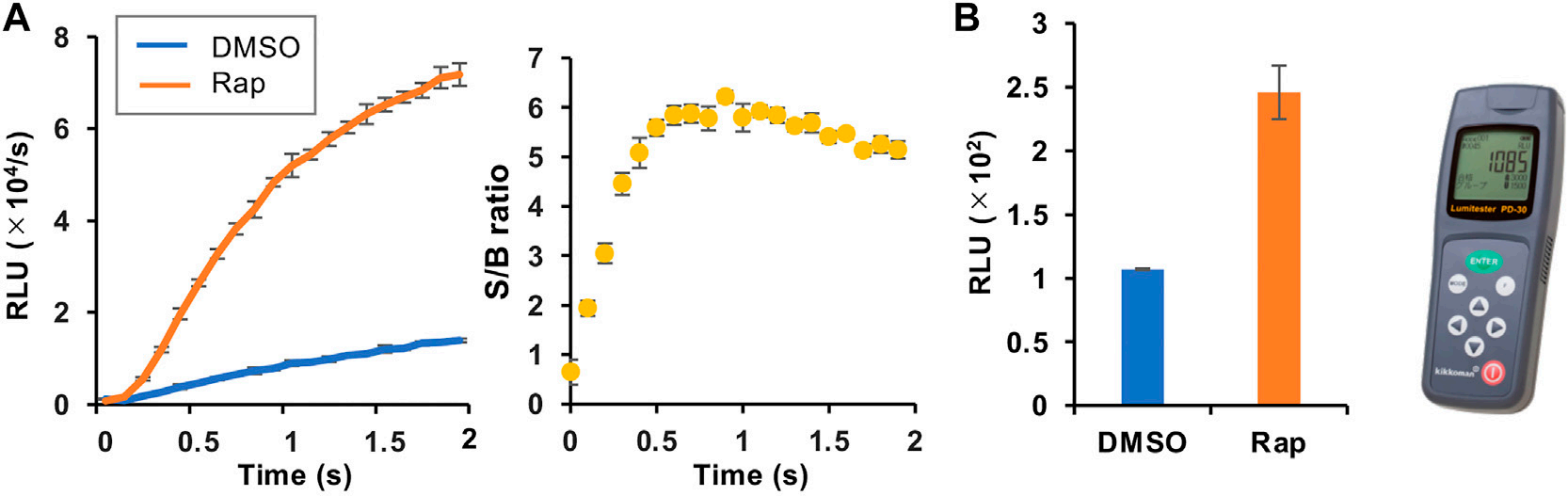
Optimization of the assay condition. **(A)** FlimPIA under the optimized condition. **(B)** Measurement using the Lumitester PD-30. Error bar: ±1 × SD (*n* = 3). Ppy Donor (50 nM) and LlL Acceptor (50 nM) were reacted with LH_2_ (33 μM) and ATP (1.25 mM) with/without rapamycin (Rap, 50 nM) in the presence of 1 mM of coenzyme A.

**FIGURE 4:**
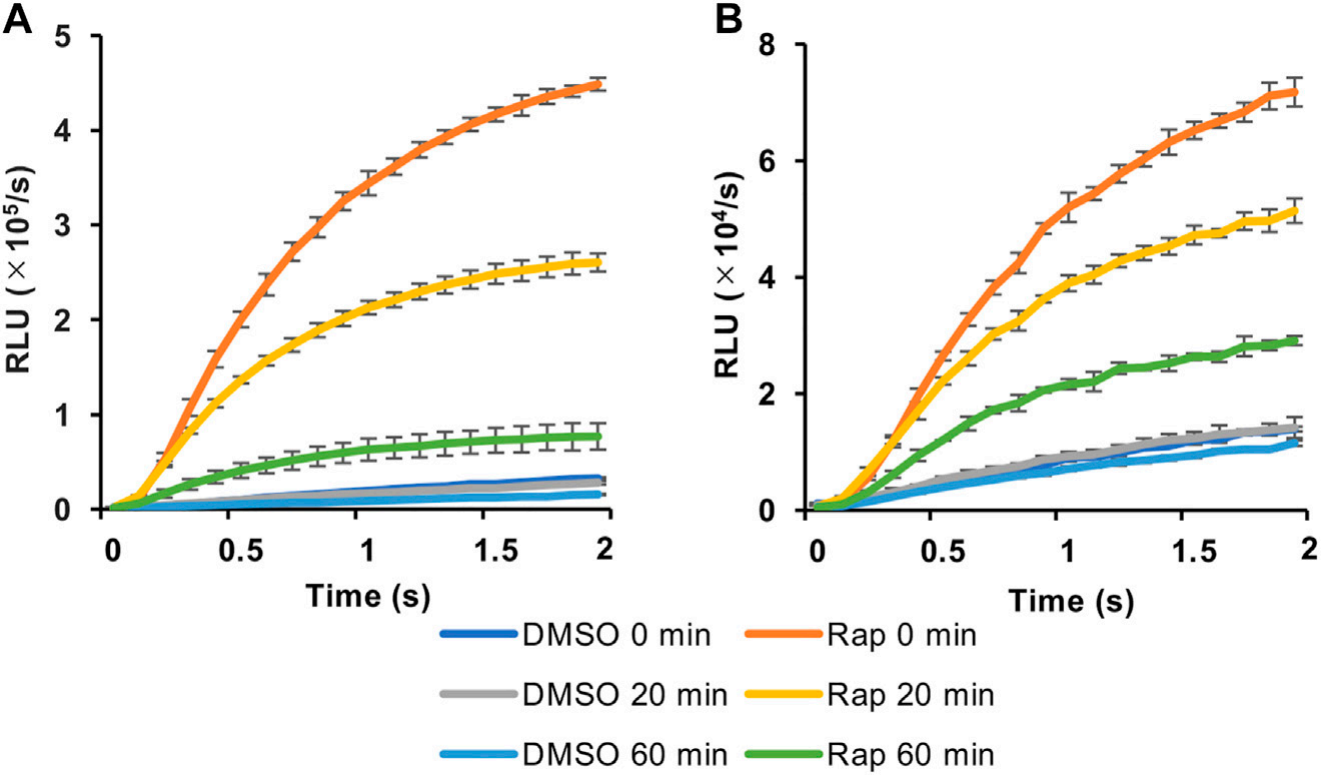
Effect of preincubation in FlimPIA. **(A)** The pair of FKBP-Ppy Donor and FRB-Ppy Acceptor. **(B)** The pair of FKBP-Ppy Donor and FRB-LlL Acceptor. After the incubation at 37°C FlimPIA was performed. Error bar: ±1 × SD (*n* = 3). Donor (50 nM) and Acceptor (50 nM) were reacted with LH_2_ (33 μM) and ATP (1.25 mM) with/without rapamycin (Rap, 50 nM). The reaction buffer was added with 1 mM of coenzyme A.

We then selected the heterologous pair of FKBP/Ppy Donor and FRB/LlL Acceptor and optimized the assay conditions. Under the conditions of 1.25 mM ATP, 33 μM LH_2,_ and 1 mM coenzyme A, the maximum S/B ratio reached more than 6 (Figure 3A). In addition, when a portable luminometer (Lumitester PD-30, Kikkoman, Chiba, Japan) was used, an S/B ratio of more than 2 was attained (Figure 3B). Finally, we compared the thermostabilities of the Ppy pair of FKBP/Ppy Donor and FRB/Ppy Acceptor and the heterologous pair of FKBP/Ppy Donor and FRB/LlL Acceptor at 37°C (Figure 4). After incubation for 20 and 60 min, the signal intensity of the Ppy pair was 58 and 17%, while the intensity of the heterologous pair was still 72 and 41%, respectively.

In conclusion, we succeeded in improving the thermostability of FlimPIA, enhancing its applicability for clinical diagnoses and inspections in such as food factories. In this study, it was revealed that both Ppy Fluc and LlL Fluc could be applied to FlimPIA. In the future, other similar enzymes such as Eluc (enhanced green-emitting luciferase) (Nakajima et al., 2010), which produces a brighter light in mammalian cells, may further expand the scope of this assay in practical applications.

## Data Availability

The original contributions presented in the study are included in the article/Supplementary Material, further inquiries can be directed to the corresponding author.
