# Supplementary material for: Improving the Stability of Protein–Protein Interaction Assay FlimPIA Using a Thermostabilized Firefly Luciferase
Source: Front Bioeng Biotechnol. 2021 Nov 11;9:778120. doi: 10.3389/fbioe.2021.778120 (PMC8631863; doi:10.3389/fbioe.2021.778120)
Supplement: Supplementary file 1 [file DataSheet1.PDF]

Supplementary Material

Supplementary Table 1. Sequence of the primers used in this study.

Primer Name	Sequence (5´- -3´)
**LlLH245D_BspHI+**	GCTATTTTAACTGTAGTACCATTTCATGATGGTTTTGGTATGTTTACTACTTTAG
**LlLH245D_BspHI+-r**	CTAAAGTAGTAAACATACCAAAACCATCATGAAATGGTACTACAGTTAAAATAGC
**LlLK443A_AseI+**	CGTGGATCGTTTGAAGTCATTAATCGCATACAAAGGATATCAAGTACCACC
**LlLK443A_AseI+-r**	GGTGGTACTTGATACCTTTGTATGCGATTAATGACTTCAAACGATCCACG
**LlLK530A_MfeI+**	GTACCTAAAGGTCTCACTGGTGCAATTGACGGTAAAGCAATTAGAG
**LlLK530A_MfiI+-r**	CTCTAATTGCTTTACCGTCAATTGCACCAGTGAGACCTTTAGGTAC
**LlL K530Q_AgeI+**	GTACCTAAAGGTCTCACCGGTCAAATTGACGGTAAAGCAATTAGAG
**LlLK530Q_AgeI+-r**	CTCTAATTGCTTTACCGTCAATTTGACCGGTGAGACCTTTAGGTAC
**LlLNotG4S-for**	GAATTCGCGGCCGCCGGTGGTGGTGGTAGCATGGAAAACATGGAGAACGATG
**LlLXho-rev**	GGAATTCCTCGAGCATCTTAGCAACTGGTTTC
**LlLS440X-KpnI-r**	ACTTGGTACCCTTTGTATTTGATTAAMWWCTTCAAACGATCCACG
**LlLS447X-KpnI-r**	CAAAGGGTACCAAGTACCACCTGCTG
**LlL530X-for**	GACGGTAAAGCAATTAGAGAAATAC
**LlLS530X-rev**	TTTCTCTAATTGCTTTACCGTCCNTTTTACCAGTGAGACC
**LlLH245X-for**	AAACATACCAAAACCMNNATGGAATGGTACTACAGTTAAA
**LlLH245X-rev**	GGTTTTGGTATGTTTACTACTTTAG
**LlLK443X-for**	TACAAAGGATATCAAGTACCA
**LlLK443X-rev**	TTGATATCCTTTGTAMNNGATTAAAGACTTCAAACGATCC
**LlLS440FW-KpnI**	ACTTGGTACCCTTTGTATTTGATTAAMMACTTCAAACGATCCACG
**LlLI530X-for**	GACGGTAAAGCAATTAGAGAAATAC
**LlLI530X-rev**	TTTCTCTAATTGCTTTACCGTCCNTTTTACCAGTGAGACC

Supplementary Table 2. Enzyme kinetics for LH_2-AMP.

	V_{max} (RLU/s)	K_m (nM)	V_{max}/K_m	R
LlL WT	2.84×10^7	53.2	5.35×10^5	0.998
LlL H245D	1.03×10^5	316	3.28×10^2	0.999
LlL K443A	7.94×10^5	193	4.10×10^3	0.999
*LlL*K529A	2.89×10^7	1210	2.38×10^4	1.000
*LlL*K529Q	2.45×10^7	562	4.36×10^4	0.999

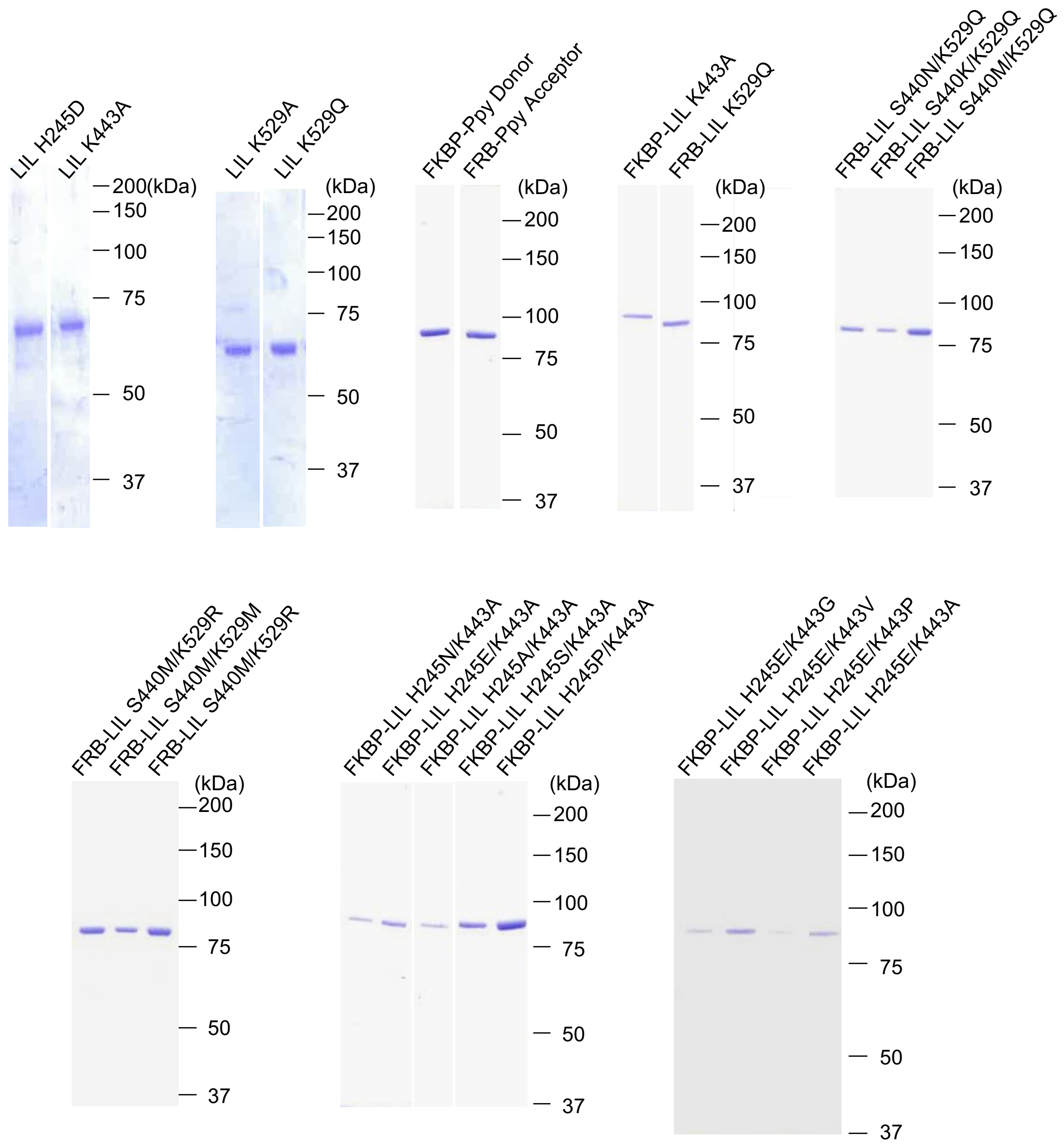

Supplementary Figure 1. SDS-PAGE of the purified proteins used for the assays.

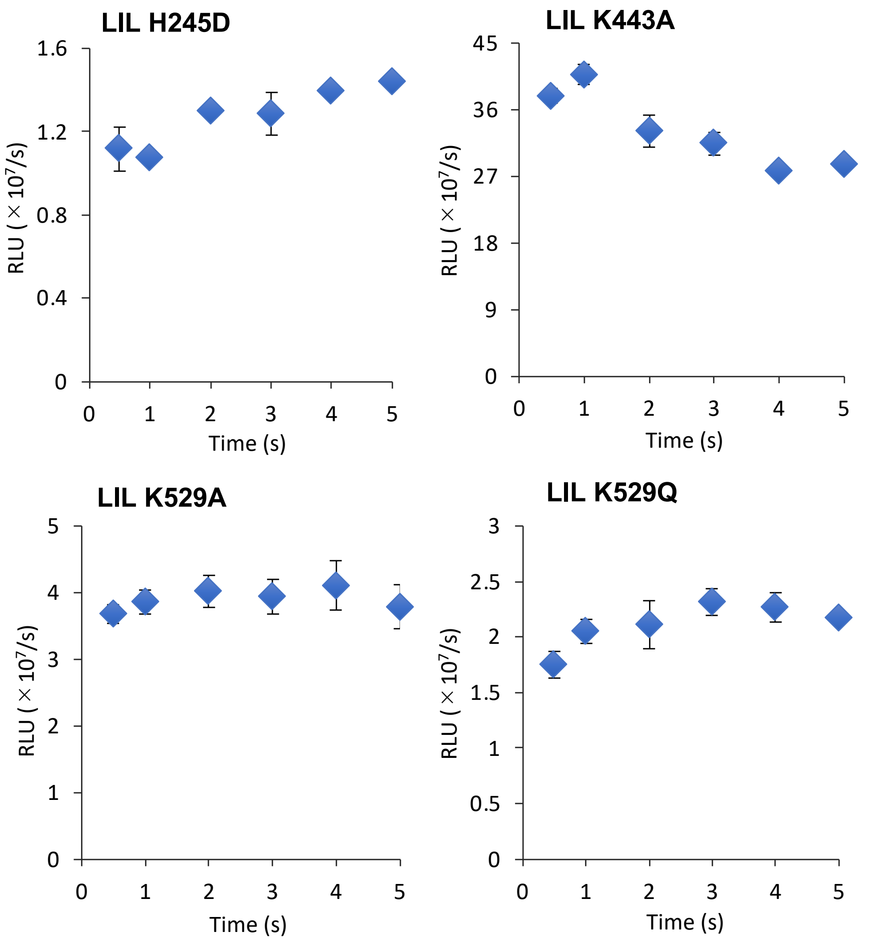

Supplementary Figure 2. Relative amounts of LH_2-AMP produced by LlL H245D, K443A, K529A and K520Q. Error bar: ±1×SD (n=3). Each enzyme (1 µM) was reacted with ATP (10 mM) and LH_2 (37.5 µM). After the reaction for 0.5, 1, 2, 3, 4 and 5 min., the reaction was stopped with HCl (0.1 N). The relative amount of LH_2-AMP produced in each reaction was estimated by adding 0.4 µM of the N-domain.

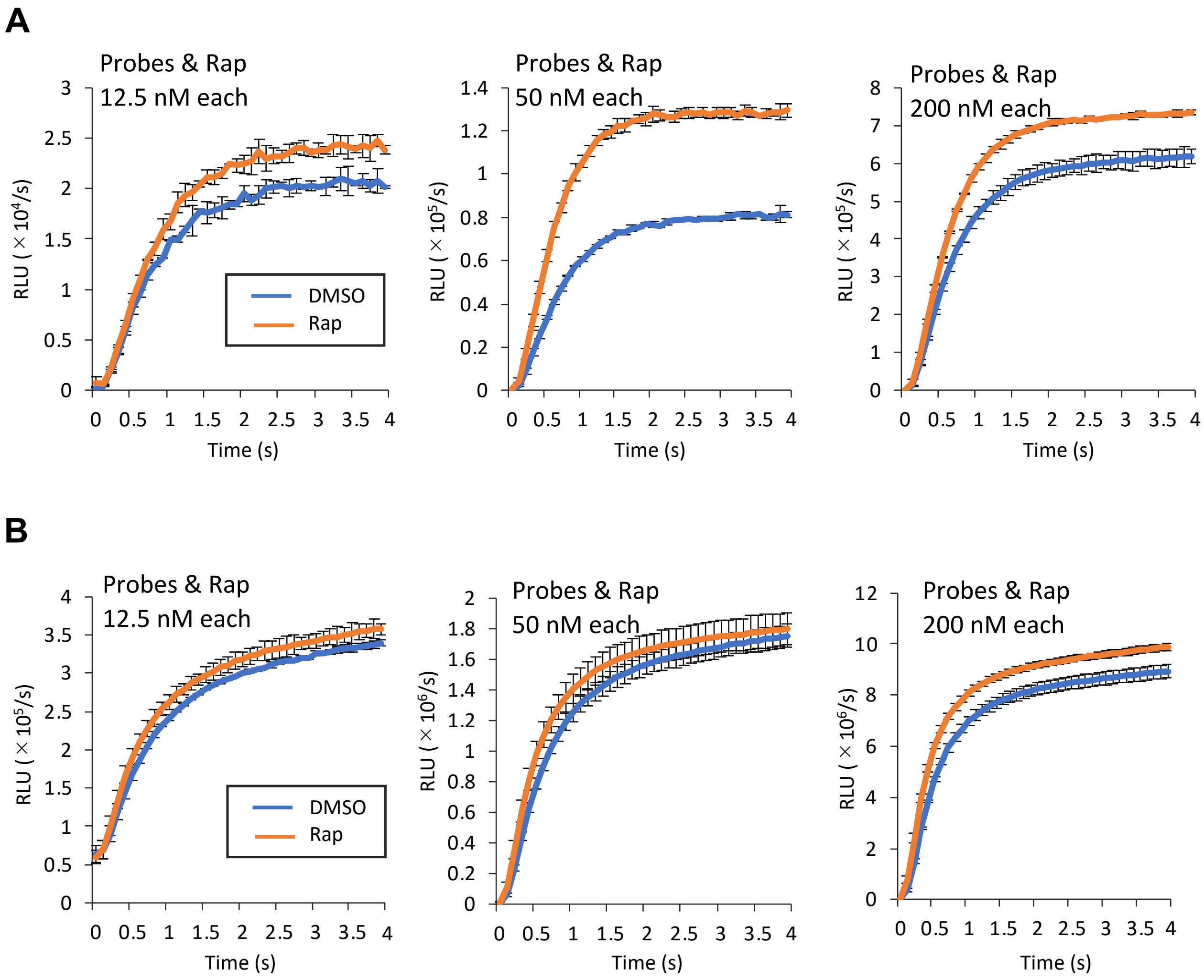

Supplementary Figure 3. FlimPIA using LlL K443A and LlL K529Q. (**A**) FlimPIA using FKBP-LlL K443A and FRB-Ppy Acceptor. (**B**) FlimPIA using FKBP- Ppy Donor and FRB-LlL K529Q. Error bar: ±1×SD (n = 3). ATP (10 mM) and LH_2 (37.5 μM) were reacted with 50 nM of the enzymes with/without 50 nM of Rap.

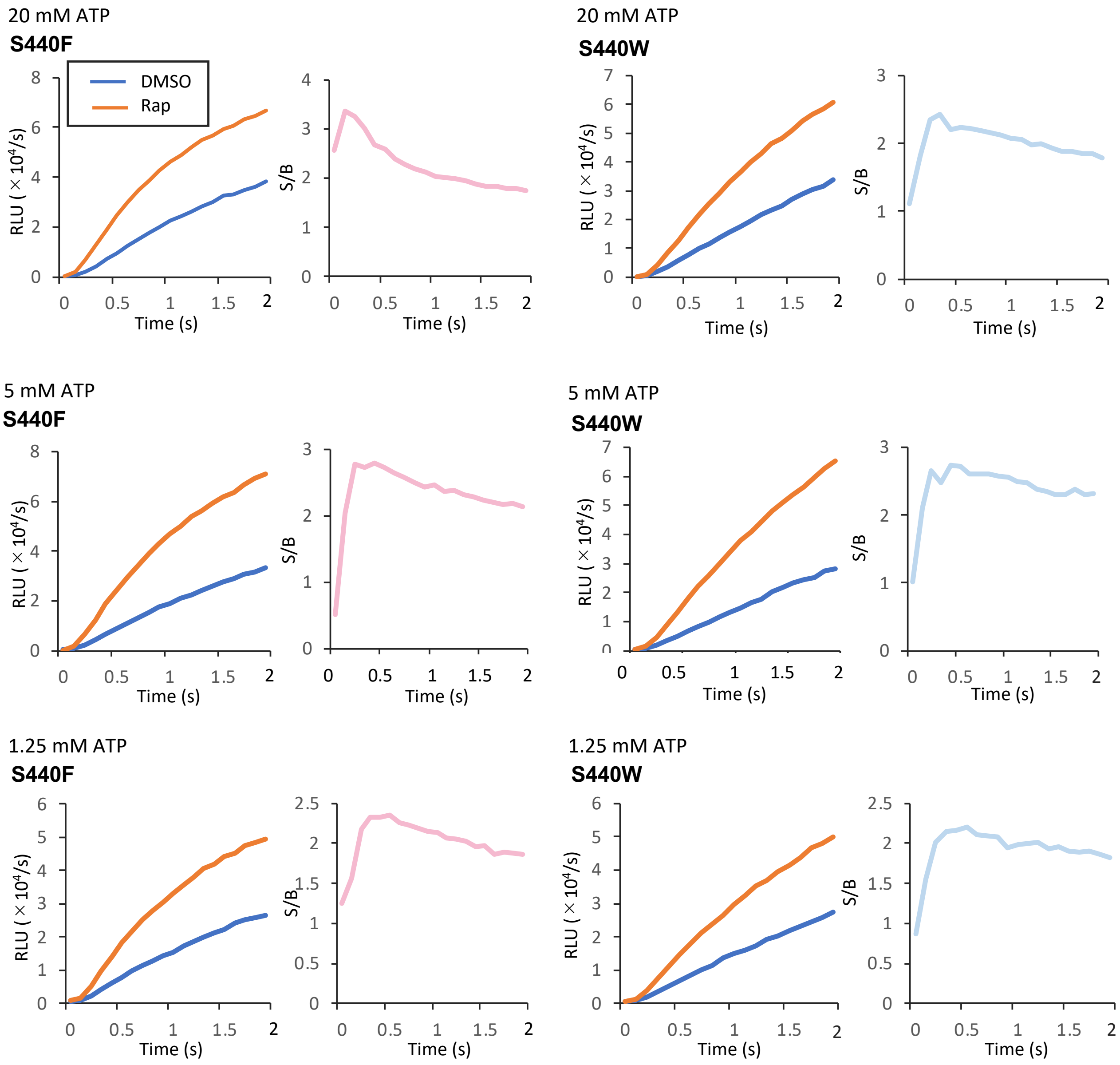

Supplementary Figure 4. FlimPIA using FRB-LIL K529Q/S440F(left) and FRB-LlL K529Q/S440W (right). Ppy Donor (50 nM) and each candidate of LlL Acceptor (50 nM) were reacted with LH_2 (37.5 nM) and ATP (20, 5, 1.25 mM) with/without Rap (50 nM).

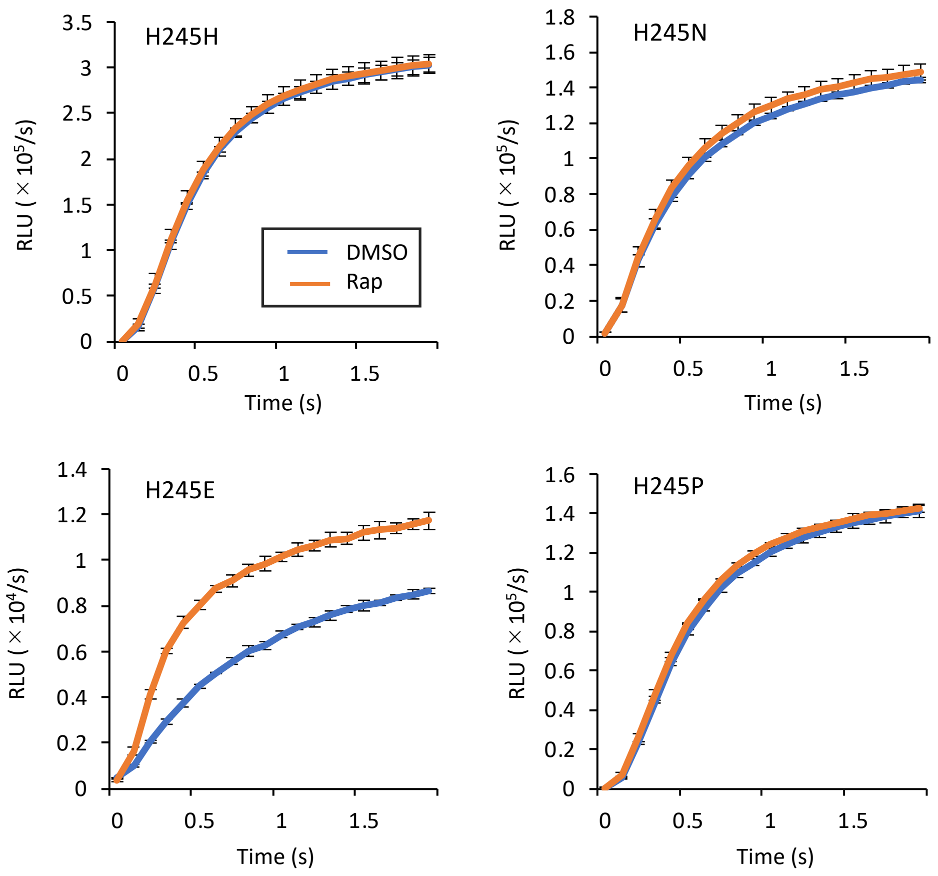

Supplementary Figure 5. Screening of H245X mutant Donors. Error bar: ±1×SD (n=3). Each candidate of LlL Donor (50 nM) and Ppy Acceptor (50 nM) were reacted with ATP (10 mM) and LH_2 (37.5 μM) with/without Rap (50 nM).

A Screening of **H245E/K443X**

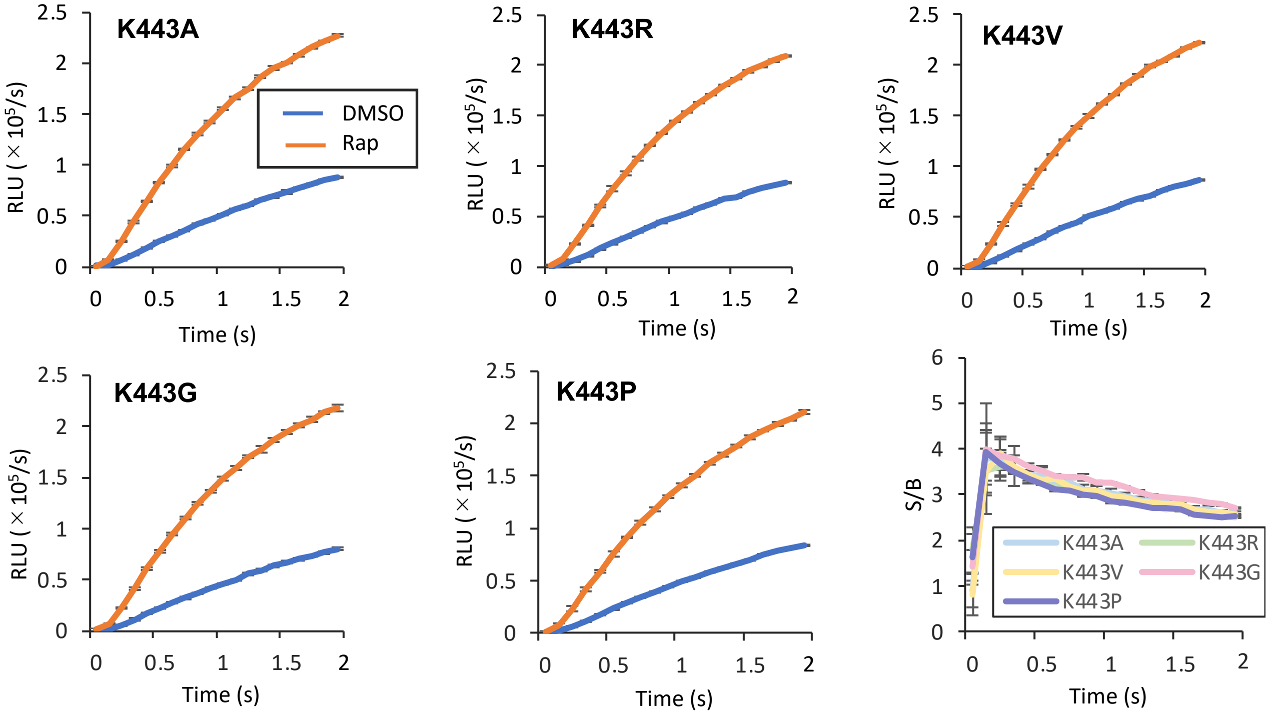

B Screening of **H245E/K443A/I530(K/R)**

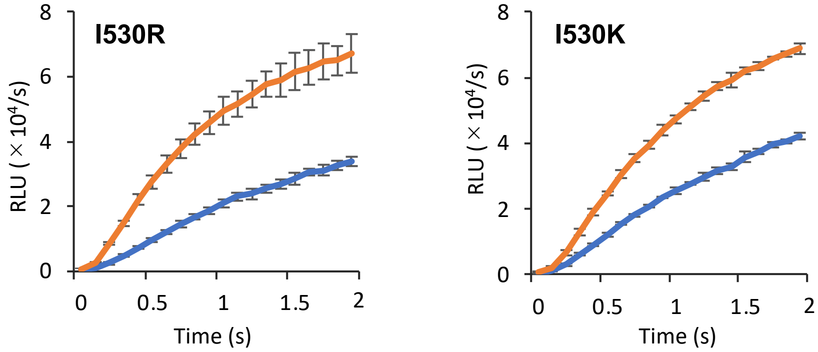

Supplementary Figure 6. Screening for LlL Donors (A) and the effect of I530R/K mutations (B). Error bar: ±1×SD (n=3). Each candidate of LlL Donor (50 nM) and Ppy Acceptor (50 nM) were reacted with ATP (10 mM) and LH_2 (37.5 µM) with/without Rap (50 nM).

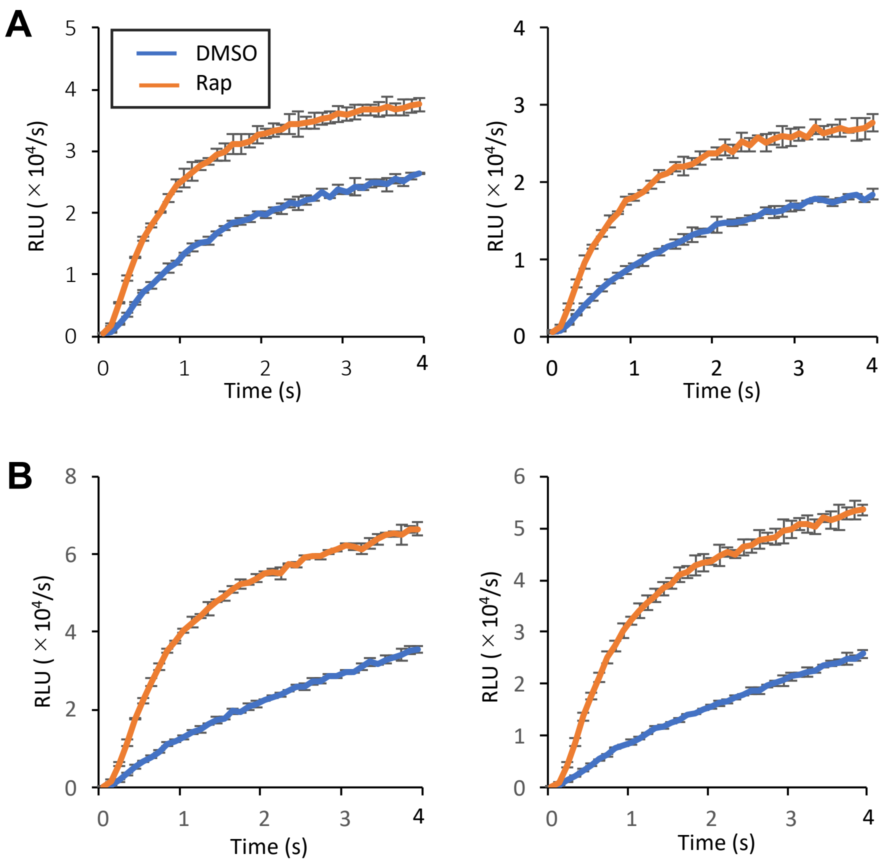

Supplementary Figure 7. Effect of long preincubation on FlimPIA. (**A**) The pair of FKBP-LlL Donor and FRB-LlL Acceptor. (**B**) The pair of FKBP-Ppy Donor and FRB-LlL Acceptor. (left) No incubation. (right) After incubation at 25°C for 2 days. Error bar: ±1×SD (n=3). Donor (50 nM) and Acceptor (50 nM) were reacted with ATP (10 mM) and LH_2 (37.5 μM) with/without Rap (50 nM).
